# Surface chemistry of quantum-sized metal nanoparticles under light illumination

**DOI:** 10.1039/d0sc04651e

**Published:** 2020-12-15

**Authors:** Shea Stewart, Qilin Wei, Yugang Sun

**Affiliations:** Department of Chemistry, Temple University 1901 North 13^th^ Street Philadelphia Pennsylvania 19122 USA ygsun@temple.edu

## Abstract

Size reduction of metal nanoparticles increases the exposure of metal surfaces significantly, favoring heterogeneous chemistry at the surface of the nanoparticles. The optical properties of metal nanoparticles, such as light absorption, also exhibit a strong dependence on their size. It is expected that there will be strong coupling of light absorption and surface chemistry when the metal nanoparticles are small enough. For instance, metal nanoparticles with sizes in the range of 2–10 nm exhibit both surface plasmon resonances, which can efficiently produce high-energy hot electrons near the surface of the nanoparticles under light illumination, and the Coulomb blockade effect, which favors electron transfer from the metal nanoparticles to the surface adsorbates. The synergy of efficient hot electron generation and electron transfer on the surface of small metal nanoparticles leads to double-faced effects: (i) surface (adsorption) chemistry influences optical absorption in the metal nanoparticles, and (ii) optical absorption in the metal nanoparticles promotes (or inhibits) surface adsorption and heterogeneous chemistry. This review article focuses on the discussion of typical quantum phenomena in metal nanoparticles of 2–10 nm in size, which are referred to as “quantum-sized metal nanoparticles”. Both theoretical and experimental examples and results are summarized to highlight the strong correlations between the optical absorption and surface chemistry for quantum-sized metal nanoparticles of various compositions. A comprehensive understanding of these correlations may shed light on achieving high-efficiency photocatalysis and photonics.

## Abstract 2

Size reduction of metal nanoparticles increases the exposure of metal surfaces significantly, favoring heterogeneous photochemistry at the surface of the nanoparticles.

## Graphical abstract

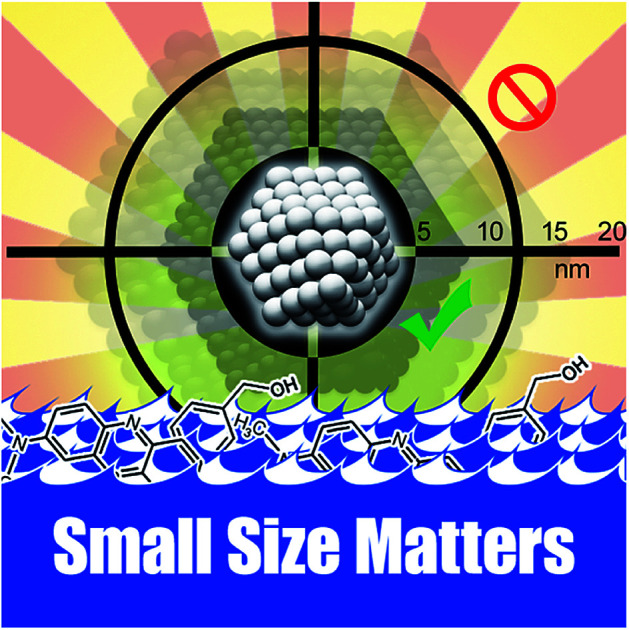

## Introduction

The use of metal nanoparticles can be traced back to Medieval Times when stained glass with varying colours became famous for decorations. Modern scientific analysis has revealed the secret that the colours originated from the presence of gold/silver nanoparticles dispersed in the stained glasses. Michael Faraday synthesized stable colloidal gold (Au) nanoparticles through chemical reduction of gold chloride with phosphorus in the 1850s, which represented one of the earliest scientific research activities on metal nanoparticles.^1^ The origin of vivid colours of the Au colloids was ascribed to the localized surface plasmon resonance (LSPR) in the nanometer-sized Au nanoparticles, which was interpreted by the Mie theory developed by Gustav Mie, a German physicist, in the early 1900s.^2^ Inspired by both the early experimental and theoretical developments, studies of colloidal metal nanoparticles with unique optical properties have been explosively reported since the late 20^th^ century. As the field has matured, many protocols have been developed to synthesize colloidal metal nanoparticles with well-controlled physical parameters and optical properties. These particles are suitable for a broad range of applications including sensing, displaying, anti-counterfeiting, biomedical imaging, therapeutics, *etc.*^3–5^

Another important class of metal nanoparticles is catalysts that have been widely explored to establish and support the chemical industry that is crucial to our society, for example, the famous Haber–Bosch process relying on iron (Fe) nanoparticles to catalyse the nitrogen fixation reaction with the promotion of ammonia production.^6^ The small sizes of metal nanoparticle catalysts are usually essential to determine their high catalytic activity by providing a large number of active surface sites. Reducing the size of metal nanoparticles offers the promise to expose high-index crystalline surface facets and surface defects (*e.g.*, steps, kinks, boundaries),^7–9^ both of which are critical to determining the chemistry of surface adsorbates and the consequent chemical reactions on/near the surfaces of the nanoparticles. In general, metal nanoparticles with smaller sizes exhibit higher catalytic activity than their counterpart particles with larger dimensions.

The long-time research efforts on metal nanoparticles have proven the strong dependence of both optical properties and catalytic activity (for surface chemistry) on the dimensions of metal nanoparticles. Such coincident dependence implies the possible strong synergy between optical properties and surface chemistry of small metal nanoparticles, indicating the potential in optically sensing surface chemistry and optically driven surface chemistry (also so-called photocatalysis). For example, light absorption in the metal nanoparticles with strong LSPR leads to the generation of hot electrons that are capable of driving redox reactions on the metal nanoparticles.^10,11^ The merging of light absorption and surface reaction in plasmonic metal nanoparticles recently triggered intensive studies of the emerging field of plasmonic catalysis, or plasmon-driven catalysis, or hot-electron-driven chemistry.^12–19^ Despite the promise and ongoing studies, the size effect on bridging the optical properties and surface chemistry of metal nanoparticles is not yet well understood. In this review, the critical length scales of nanoparticles made of metal elements will be reviewed to highlight the appropriate size range in which the metal nanoparticles can effectively synergize light absorption and surface chemistry. In this size range, the metal nanoparticles are defined as “quantum-sized metal nanoparticles”. The strong influence of surface chemistry on optical absorption, as well as the dependence of surface chemistry on optical absorption, will be comprehensively discussed with the assistance of typical examples of “quantum-sized metal nanoparticles”. This article will end with personal remarks that will discuss the challenges of current research and future directions.

## Quantum-sized metal nanoparticles

**Fig. 1:**
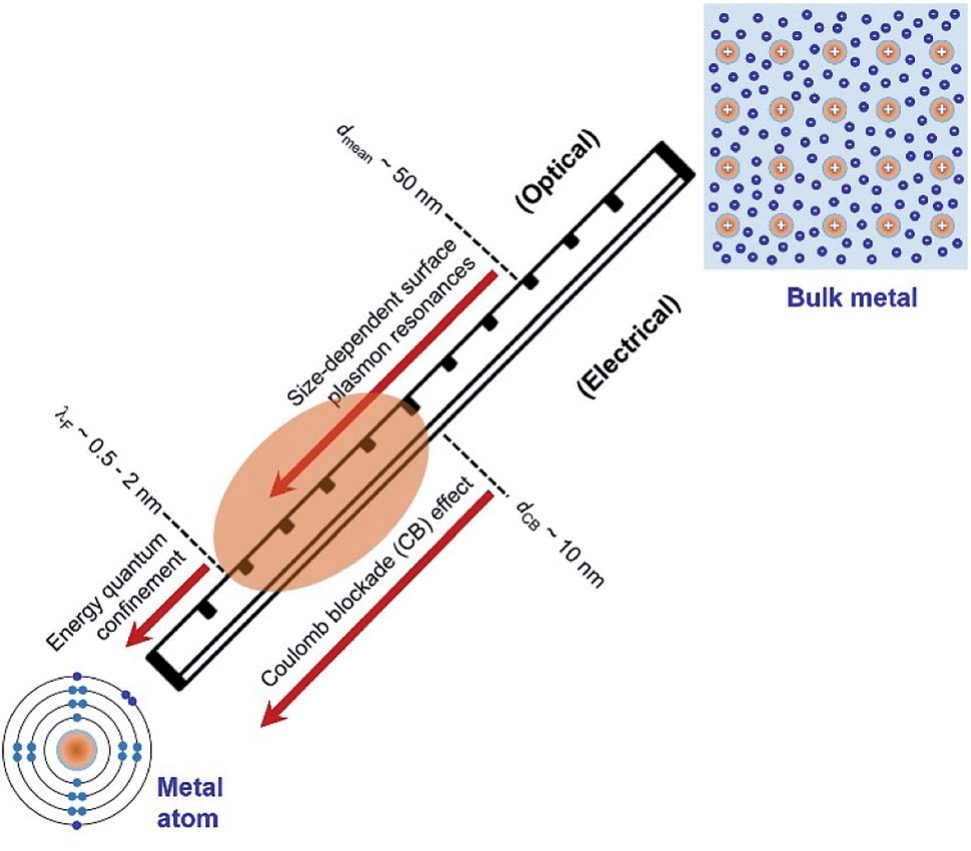
Critical length scales of nanoparticles made of metal atoms in the range of exhibiting unique quantum phenomena that determine the optical and electrical properties of the nanoparticles. The nanoparticles with sizes in the range of *λ*_F_ − *d*_CB_ (highlighted by the orange oval) represent the focus of this review, which are presented as “Quantum-Sized Metal Nanoparticles” (or QSMNPs). The QSMNPs exhibit both plasmon-based light absorption and surface Coulomb blockade effect. (Bottom left) Configuration of electrons in a single atom of metal element with inner-shell electrons (light blue) and outer-shell valence electrons (blue). (Top right) Configuration of fee electrons in a solid made of many atoms of metal element, highlighting the existence of free electrons (blue) that originate from the outer-shell electrons of individual atoms.

Electrons in individual atoms behave similarly regardless of the type of elements, *i.e.*, electrons occupy discrete orbitals with well-separated energy levels (bottom left, Fig. 1). The behaviour of electrons starts to be significantly different when a large number of atoms condense into solids, differentiating the solids into metals, semiconductors, and insulators. According to band theory, the valence band filled with valence electrons and the empty conduction band are continuous or overlapped with the valence band in a metal solid, whereas the valence band and the conduction band are well separated by an energy gap in a semiconductor solid or insulator solid. The continuity and overlap of the energy states in the valence band and conduction band in a metal solid allow the valence electrons to move freely without suffering from an obvious energy barrier. From this standpoint, a solid is called a “metal” only when the valence electrons in the solid can move freely like “free electrons”. In contrast, a solid with valence electrons confined at specific energy levels cannot be called a “metal” even though the solid is formed from the condensation of atoms of metal elements. For instance, a nanoparticle with a small enough size is composed of only a few atoms of a metal element, and the density of energy levels (*i.e.*, states) in both the valence band and conduction band are well separated with energy gaps more significant than the thermal energy. The discreteness of the energy levels leads to opening a HOMO–LUMO gap, resulting in molecular-like optical properties. Such energy quantization effects within a nanoparticle made of metal elements occur when the nanoparticle size is comparable to (and smaller than) the electron's de Broglie wavelength (or Fermi wavelength, *λ*_F_) (Fig. 1). The energy quantization effects have been witnessed by the appearance of multiple molecular-like optical absorption peaks and light emissions for nanoclusters made of few atoms of metal elements that exhibit diameters in the range of 0.5–2 nm.^20–23^ It is worth pointing out that the energy quantization effects in the nanoclusters prevent the free movement of valence electrons (*i.e.*, one of the characteristic properties of metals).

As the size of nanoparticles is larger than the electron's de Broglie wavelength, the density of states becomes high enough to eliminate the energy barrier of freely moving electrons from the valence band to the conduction band. The availability of free electrons enables the nanoparticles to behave as a metallic system containing Fermi electron gas. Therefore, it is more reasonable to describe the nanoparticles made of metal elements with sizes larger than 2 nm as “metal nanoparticles”. The free electrons in a metal nanoparticle can respond to an electromagnetic wave and oscillate upon illumination of light with an appropriate wavelength. Quantization of the collective oscillations of the free electrons is described as plasmons, *i.e.*, one type of quasiparticle. When the size of a metal nanoparticle is less than the mean free path of electrons, *d*_mean_, the oscillation movement of the electrons will interfere with the nanoparticle surface. Such surface interference changes the dielectric function of the metal nanoparticle to modulate the resonant oscillation frequency of the electrons depending on the size of the nanoparticle. The corresponding resonant oscillations are LSPR that are responsible for the strong light absorption in the metal nanoparticle (Fig. 1). The light absorption power is proportional to the volume of the nanoparticle. The photoexcited metal nanoparticle then undergoes non-radiative relaxations (*e.g.*, Landau damping) to generate so-called “hot electrons” with much higher kinetic energies near the nanoparticle surface and “lukewarm electrons” with slightly increased kinetic energies within the nanoparticle.

It is well known that electrons represent one typical class of reactive species to be involved in a broad range of chemical reactions. The hot electrons generated near the surface of photoexcited metal nanoparticles can be self-injected into orbitals of the adsorbates on the surfaces of the nanoparticles, leading to the change of reactivity of the surface adsorbates. On the other hand, the capture of electrons in the surface adsorbates influences the optical properties of the nanoparticles. The electron flow from the metal nanoparticles to surface adsorbates, *i.e.*, an electrical property of the metal nanoparticles, depends on the size of the nanoparticles. The dependence becomes predominant for the metal nanoparticles with sizes less than 10 nm at which the strong Coulomb blockade (CB) effect emerges (Fig. 1).^24^ The Coulomb blockade effect describes that the charging energy (*U*) required to inject an additional electron into a metal nanoparticle increases inversely with the nanoparticle size by following1
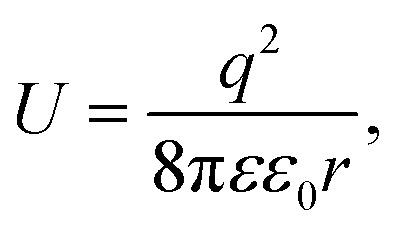
where *q* and *ε*_0_ are the electron charge and the permittivity of free space, respectively, *ε* and *r* are the relative dielectric constant and the radius of the metal nanoparticle. The inverse proportionality between *U* and *r*, on the other hand, implies that the electrons in small metal nanoparticles suffer a low energy barrier to emit (or scatter) out compared to the counterpart large nanoparticles. Such a “reverse Coulomb blockade effect” indicates that reducing the size of metal nanoparticles benefits electron injection from the metal nanoparticles to surface adsorbates. Moreover, hot electrons generated in small metal nanoparticles experience a short travel length, usually shorter than their inelastic mean free path, to reach the metal/adsorbate interface. As the size of metal nanoparticles decreases, the travel length of hot electrons reduces accordingly. The shorter travel length of hot electrons lowers the consumption of kinetic energy when the hot electrons reach the metal/adsorbate interface, leaving a larger fraction of energy to facilitate the electron injection from the metal surface to adsorbate species and/or to promote the direct interaction between the hot electrons with the adsorbate species.^25,26^ The effect of shorter travel length of hot electrons in smaller metal nanoparticles and significant “reverse Coulomb blockade effect” on the surface of smaller metal nanoparticles can synergistically benefit surface chemical reactions, resulting in the observation of faster reaction kinetics on smaller metal nanoparticles.

Fig. 1 summarizes the three critical lengths (*i.e.*, *λ*_F_, *d*_mean_, and *d*_CB_) of nanoparticles made of metal elements, below which the optical and electrical properties of the nanoparticles become size-dependent quantum phenomena. When the size of nanoparticles falls between *λ*_F_ and *d*_CB_, both the optical and electrical properties of the nanoparticles strongly depend on their size. In the size range of *λ*_F_ − *d*_CB_ as shaded by the orange ellipse in Fig. 1, the valence electrons in the nanoparticles can move freely to behave similarly to those in metals. In this review, the nanoparticles made of metal elements with size in the range of *λ*_F_ − *d*_CB_ (*i.e.*, 2–10 nm) are named as “Quantum-Sized Metal Nanoparticles” (or QSMNPs), which exhibit both LSPR (quantum optical properties) and a strong Coulomb blockade effect (quantum electrical properties).

Under the conditions of LSPR, the electric displacement field (**D**) and the incident electric field (**E**) follow a relationship

2 **D**(**k**,*ω*) = *ε*_0_*ε*(**k**,*ω*)**E**(**k**,*ω*).

**Fig. 2:**
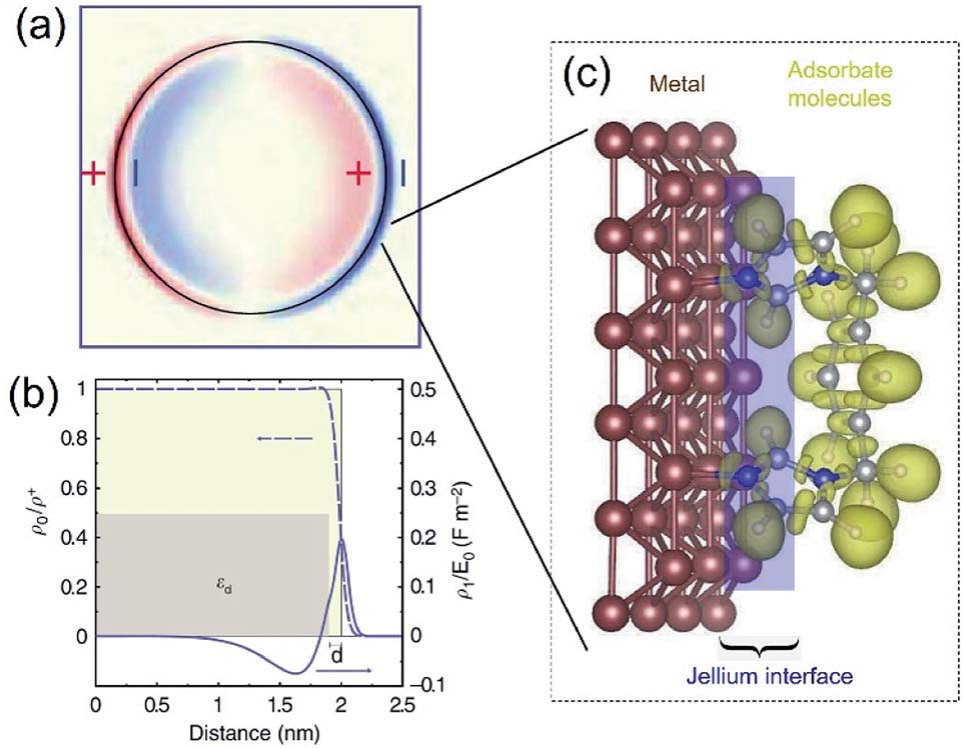
(a) The calculated spatial distribution map of induced charge density [*ρ*_1_(**r**)] of the cross section of a silver cylindrical nanowire with a diameter of 4 nm under the illumination of light at the respective dipolar LSPR frequency of 3.594 eV. The calculations were based on the self-consistent hydrodynamic model (SC-HDM) that is more accurate for describing the inhomogeneous electron gas. (b) Profiles of equilibrium electron charge density *ρ*_0_ normalized to the ion charge density *ρ*^+^ (dashed line) and induced charge density *ρ*_1_ normalized to the perturbing field amplitude *E*_0_ for the dipolar LSPR, as a function of the distance from the center of the nanowire. The light yellow shaded area indicates the jellium background profile. The grey shaded area shows the extension of the effective polarizable medium describing the optical response of the 4d core electrons. (c) Schematic illustration of the metal/adsorbate interface (highlighted by the purple shaded area) at which free electrons in the metal are redistributed among the metal lattice and the adsorbate species. (a and b) Adapted from ref. 28, Macmillan Publishers Limited Copyright 2015.

The dielectric constant *ε*(**k**,*ω*) of a QSMNP is a function of both the wave vector **k** and angular frequency *ω* of the incident light. Fourier transform of eqn (2) to the spatial domain leads to a spatially nonlocal relationship between **D** and **E** because of the **k** dependence in *ε*(**k**,*ω*). The nonlocal effect is too significant in QSMNPs with sizes of <10 nm to be ignored even in theoretical calculations of the optical properties of the QSMNPs.^27^ For example, Fig. 2a shows the calculated charge density distribution *ρ*_1_(**r**) in the cross-section of a Ag nanowire with a radius of 2 nm at the LSPR frequency of 3.594 eV.^28^ The calculations rely on the introduction of a self-consistent hydrodynamic Drude model (SC-HDM) for the inhomogeneous electron gas in the Ag nanowire. The corresponding LSPR absorption spectrum of the Ag nanowire is more accurate and consistent with the experimental observations. The presence of charges outside the physical wall of the Ag nanowire indicates the spill-out of the electron density from the metal to the surrounding medium, which is against the “hard wall model” involving the spill-in of the electron density inside the metal. Fig. 2b presents the equilibrium electron charge density *ρ*_0_ normalized against the metal cation charge density *ρ*^+^ as a function of the distance to the centre of the nanowire cross-section (dashed curve). The non-uniform spatial electron density near the surface of the nanowire highlights the charge spill-out effect and onset of Friedel oscillations. The light-induced net charge density *ρ*_1_ exists up to a distance away from the jellium interface, resulting in an oscillation in the internal region and a peak followed by a decay in the free-space region (solid curve). The nonlocal effect or spill-out effect of electrons in small metal nanoparticles, *e.g.*, QSMNPs, leads to the presence of electrons beyond the physical surface of the nanoparticles under the illumination of light with appropriate wavelengths. The spilled-out electrons can actively interact with adsorbate species in the proximity of the nanoparticle surfaces (Fig. 2c). The electron–adsorbate interactions perturb the electron oscillations under light illumination, leading to a change of light absorption properties of metal nanoparticles. On the other hand, the electron–adsorbate interactions may weaken the strength of chemical bonds to activate the adsorbate species, promoting chemical reactions on the nanoparticle surfaces. Therefore, light absorption properties and surface chemistry become strongly interdependent for QSMNPs because of the strong nonlocal effect of free electrons and strong inverse Coulomb blockade effect in metal nanoparticles smaller than 10 nm.

## Influence of surface chemistry on optical absorption of QSMNPs

**Fig. 3:**
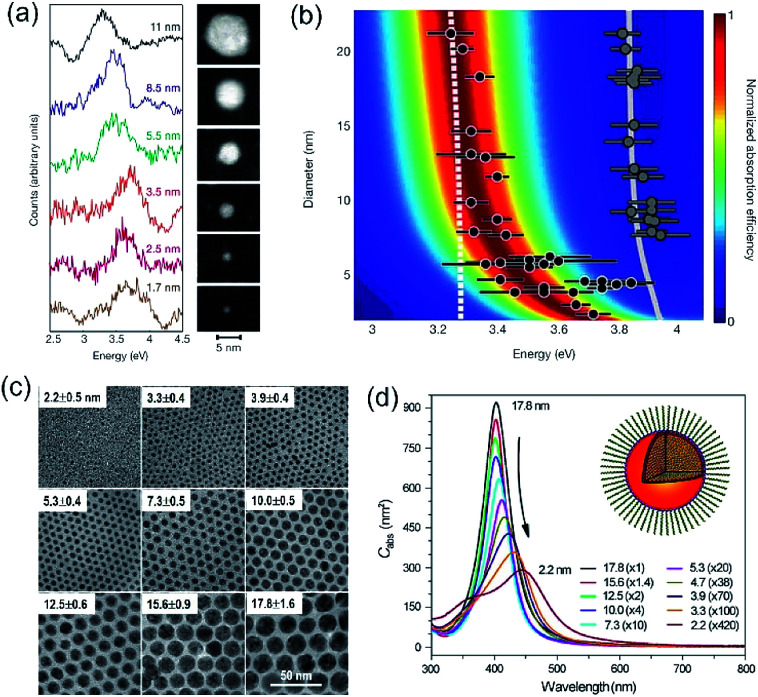
(a and b) LSPR spectra of Ag nanoparticles with clean surfaces and varying sizes. (a) LSPR absorption spectra deconvoluted from EELS data of individual Ag nanoparticles shown in the right TEM images. The values associated with spectra refer to the diameters of the corresponding nanoparticles. (b) Theoretical calculations of LSPR energies of Ag nanoparticles with varying diameters using the DFT-derived permittivity model. The black dots represent the experimentally measured LSPR energies of individual Ag nanoparticles derived from the EELS data. The predictions calculated with the classical Mie theory are also presented for reference (dashed line). The gray dots and gray line on the right part of the panel are the experimentally measured and theoretically calculated bulk resonance energies of Ag nanoparticles with different sizes. (a and b) Adapted from ref. 29, Macmillan Publishers Limited Copyright 2012. (c and d) Characterization of differently sized Ag nanoparticles coated with oleylamine molecules. (c) TEM images and (d) averaged absorption spectra of single nanoparticles. The numbers out of the parentheses represent the diameters of the Ag nanoparticles and the numbers in the parentheses are the amplifications of absorption cross sections. The inset shows the representative model of a Ag nanoparticle coated with oleylamine molecules. (c and d) Adapted from ref. 30, PNAS Copyright 2010.

Accurately measuring and comparing the LSPR absorption spectra of QSMNPs with different sizes is challenging because the non-uniformity of individual nanoparticles in ensemble samples can significantly broaden the absorption peaks to smear the size-dependent characteristic peaks. This challenge has been solved by using aberration-corrected transmission electron microscopy (TEM) imaging and monochromated scanning TEM (STEM) electron energy-loss spectroscopy (EELS), which can precisely determine the sizes and LSPR absorption spectra of individual QSMNPs, respectively.^29^ For example, Scholl *et al.* synthesized Ag nanoparticles through direct reduction of AgNO_3_ with NaBH_4_ in an aqueous solution, forming a sample containing Ag nanoparticles with clean surfaces (*i.e.*, no coating of surfactant molecules) that exhibited a broad distribution of diameters ranging from 20 nm down to 1.7 nm. Fig. 3a presents the STEM images and EELS spectra of individual Ag nanoparticles with varying diameters, *i.e.*, 11 nm, 8.5 nm, 5.5 nm, 3.5 nm, 2.5 nm, and 1.7 nm. The EELS spectra were recorded by directing the electron beam at the edge of the nanoparticles to excite the LSPR selectively. The peak width of the LSPR spectra increases with the decrease of the nanoparticle size, with a full-width at half-maximum (FWHM) increasing from 0.4 eV to 0.6 eV as the diameter of the Ag nanoparticles decreases from 11 nm to 1.7 nm. The LSPR peak position (*i.e.*, the energy of surface plasmon resonance) exhibits a remarkable blueshift (*i.e.*, energy increase from 3.3 eV to 3.8 eV) as the nanoparticle size decreases (black dots, Fig. 3b). In contrast, classical Mie theory would predict a maximum blueshift of only 0.03 eV over the entire size range shown in Fig. 3b (dashed white line). The measured LSPR peak positions are close to those of the Mie theory prediction for the Ag nanoparticles with diameters larger than 10 nm. However, the consistency becomes invalid as the Ag nanoparticles are smaller than 10 nm, confirming the occurrence of the size-dependent quantum effect that strongly influences the LSPR of the Ag QSMNPs. By incorporating the quantum theory (including the electron spill-out effect), the calculated LSPR absorption spectra of the Ag QSMNPs agree well with the EELS spectra. Fig. 3b presents the calculated LSPR spectra of Ag nanoparticles with different sizes based on the density functional theory (DFT)-derived permittivity mode. It is worth pointing out that the trend of the LSPR absorption peak position toward higher energy is not purely monotonic in the EELS data, with a greater variety in peak positions occurring at the smallest sizes. Such significant variations of peak positions indicate that the tiny metal nanoparticles are very sensitive to the non-uniformity of individual nanoparticles in terms of geometry and surface conditions due to the strong quantum effect.^20^

As mentioned in the previous section and Fig. 2, the surface chemistry of QSMNPs could change the electron distribution near the surface of the QSMNPs, highlighting the potential to influence the optical properties of the QSMNPs. Fig. 3c presents the TEM images of the Ag nanoparticles of various sizes in the range of 2–18 nm synthesized through the reduction of AgNO_3_ in oleylamine at elevated temperatures.^30^ The surfaces of the resulting Ag nanoparticles are covered with oleylamine molecules (inset, Fig. 3d) that serve as surfactant capping reagents to stabilize the Ag nanoparticles and ensure the high uniformity of the Ag nanoparticles. The average LSPR absorption spectra of these Ag nanoparticles dispersed in hexane are presented in Fig. 3d, highlighting the dependence of the LSPR absorption of the Ag nanoparticles on their diameter. As particle size decreases, the LSPR peak height dramatically decreases and the peak width gradually becomes broader. The dependence of the absorption peak position on the nanoparticle size is significantly different from those nanoparticles without a surfactant coating shown in Fig. 3a. The absorption peak slightly blueshifts as the particle diameter decreases from ∼18 nm to ∼12 nm, and then strongly redshifts as the particle diameter further decreases down to ∼2 nm (Fig. 3d). Comparing the results of Fig. 3b and d shows a significant difference in the size-dependence LSPR absorption peak positions between the Ag nanoparticles with clean surfaces and the Ag nanoparticles with the oleylamine coating, in particular when the Ag nanoparticles are smaller than 10 nm. As particle size decreases, the LSPR absorption peak of the oleylamine-coated Ag QSMNPs redshifts, whereas the absorption peak of the clean Ag QSMNPs blueshifts. The comparison shown in Fig. 3 represents a typical example highlighting that the chemistry of surface adsorbates is crucial to determine the optical absorption of QSMNPs with sizes of 2–10 nm. Immobilizing electrons in surface adsorbates decreases the density of free electrons in the QSMNPs, leading to the weakened intensity and lowered frequency of the LSPR. The influence becomes severe as the size of the QSMNPs decreases.^30^

The influence of forming Ag–N(oleylamine) adsorption bonds on the LSPR absorption of the colloidal Ag nanoparticles can also be interpreted by the concept of “chemical interfacial damping” (CID). The Ag–N adsorption bond provides additional energy states near the Ag nanoparticles' surface to allow the decay of plasmons excited in the Ag nanoparticles, representing an important plasmon damping channel for QSMNPs with chemically modified surfaces. The presence of CID lowers the intensity and broadens the width of LSPR peaks of plasmonic nanoparticles. The CID also influences the position of LSPR peaks (corresponding to the resonance frequencies), which usually shifts to the red with strong CID. The occurrence of CID has been evidenced by two-photon photoemission (2PPE) spectroscopy.^31^ In a microscopic picture, the surface adsorbates induce inside the metal electric dipoles that act as additional scattering centers for plasmon dephasing.^32^ The studies of homogeneous plasmon line width of Au nanorods reveal that CID scales inversely with the effective path length of electrons, *i.e.*, the average distance of electrons to the nanorod surface.^33^ The contribution of CID to the plasmon energy decay becomes more dominant as the size of plasmonic metal nanoparticles decreases.

## Generation of hot electrons in photo-excited QSMNPs

**Fig. 4:**
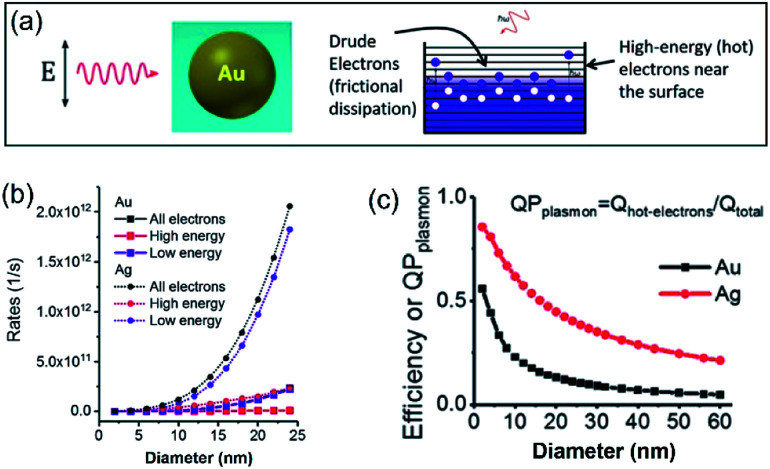
(a) Illustration of the Fermi electron sea with excited electrons and holes in a photoexcited Au nanosphere. The excited electron–hole pairs in the bulk (*i.e.*, inside the Au nanoparticle) have small energies, whereas the carriers (including both electrons and holes) generated near the surface are more energetic. (b) Calculated generation rates of Drude electrons (blue symbols) and high-energy hot electrons (red symbols) for spherical Au and Ag nanoparticles with varying diameters. (c) Energy efficiency of generating hot electrons in spherical Au and Ag nanoparticles with varying diameters. In the calculations, the excitation frequencies for Au nanoparticles and Ag nanoparticles were 2.36 eV and 3.12 eV, respectively. The intensity of the incident light was 3.6 × 10^3^ W cm^−2^. Adapted from ref. 12, American Chemical Society Copyright 2017.

In a photo-excited metal nanoparticle, the sequential decay yields excited electron–hole pairs followed by generating energetic electrons with a broad range of energies.^34,35^ For example, the LSPR decay in a Au nanoparticle involves several different processes: (i) direct bulk-like decay into electronic and phononic excitations, (ii) radiation damping to scatter a photon, and (iii) damping due to electron–surface collisions (Fig. 4a).^36–39^ The radiation damping process (ii) is not responsible for the energy elevation of electrons. The direct bulk-like decay process (i) contains contributions from interband and intraband (“Drude model”) transitions, generating excited electrons with energy slightly above the Fermi level that are called Drude electrons (or lukewarm electrons). The surface term (iii) is responsible for the generation of hot electrons with energy far above the Fermi level. The generation rate of Drude electrons is proportional to the volume of the Au nanoparticles, while the generation rate of hot electrons is proportional to the diameter of the Au nanoparticles.^35,40–42^ The nanoradiative decay of plasmons in the photo-excited Au nanoparticles results in the non-uniform redistribution of electrons in both energy and spatial dimensions, *i.e.*, lukewarm electrons in the body of the nanoparticles and high-energy hot electrons near the surface of the nanoparticles. The degree of distribution of non-uniformity increases with the decrease of the nanoparticle size. Fig. 4b plots the dependence of the energetic electron generation rate on the diameter of Au nanoparticles under photo-illumination, showing that the generation rates of both low-energy Drude electrons and high-energy hot electrons increase with the size of the Au nanoparticles. The Au nanoparticles with larger sizes favour the generation of Drude electrons, while the generation of hot electrons dominates for the Au nanoparticles with smaller sizes. The generation of energetic electrons in Ag nanoparticles exhibits similar size-dependent rates to the Au nanoparticles (Fig. 4b).

The size-dependent generation rates of Drude electrons and hot electrons plotted in Fig. 4b are consistent with the fact that the generation of hot electrons is a surface phenomenon while the generation of Drude electrons represents a bulk effect. The high-energy hot electrons generated near the nanoparticle surfaces are usually reactive toward the adsorbate species (if any) on the nanoparticle surfaces, potentially triggering chemical reactions. Therefore, the energy efficiency of generating hot electrons, Eff_hot-electrons_, represents an important parameter for evaluating the photo-to-chemical energy conversion efficiency in photocatalysis. The value of Eff_hot-electrons_ (or quantum yield of producing hot electrons from the plasmon, QP_plasmon_) increases with the decrease of the particle size:3
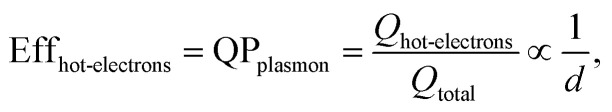
where *Q*_hot-electrons_ and *Q*_total_ are the light absorption responsible for the generation of hot electrons and the total light absorption in a metal nanoparticle, respectively, and *d* represents the diameter of the nanoparticle. The hyperbolic relationships shown in Fig. 4c confirm that QSMNPs with sizes of less than 10 nm are more efficient in generating high-energy hot electrons upon light absorption. For example, the energy efficiency for Ag nanoparticles with a diameter of 6 nm is 0.72, which is 1.31 times of that for Ag nanoparticles with a diameter of 13 nm (*i.e.*, 0.55).

## Hot-electron-driven surface chemistry of QSMNPs

The photo-excited high-energy hot electrons concentrated near the surface of QSMNPs could be injected into the empty (antibonding) orbitals of adsorbate molecules to elevate the potential energy of the adsorbate molecules.^10,14^ The positive charges left behind in the QSMNPs are “hot holes”. The uplifted potential energy activates the adsorbate molecules to promote chemical reactions. In other words, the filling of hot electrons in the antibonding orbitals weakens the strength of the corresponding chemical bonds to facilitate the cleavage of the bonds, favouring (or enabling) chemical reactions. Such chemical reactions enabled by hot electrons that mediate energy transfer from photons to reactant molecules are called “hot-electron-driven surface chemistry”.^43–45^ After the activation of the reactant molecules, the hot electrons lose energy to become cool ones, which follows two possible relaxation pathways. If the cool electrons flow back to the QSMNPs, they recombine with the hot holes to neutralize the charges, recovering the nanoparticles. If the cool electrons preferably remain in the reactant molecules, highly reactive radical anions are formed to trigger consequential reactions. Meanwhile, the hot holes left in the QSMNPs are capable of driving oxidation reactions on the nanoparticle surfaces to neutralize the charges in the QSMNPs, which are called “hot-hole-driven surface chemistry”.^14^ In both pathways, the QSMNPs behave unchanged, consistent with the characteristics of a catalyst that mediates energy input of light illumination to drive chemical reactions. In general, hot electrons are more mobile than the complementary hot holes, leading to the injection of hot electrons into the target chemical bonds much faster than the hot holes traveling to the target adsorbate molecules. Therefore, hot-electron-driven surface chemistry plays the predominant role even through hot-hole-driven surface chemistry could contribute to the overall photocatalytic reactions because hot-hole-driven surface chemistry can only possibly occur after the consumption of the hot electrons in the hot-electron-driven surface chemistry.

The results shown in Fig. 4 indicate that more hot electrons are expected to generate in small QSMNPs and to concentrate on the surface of the QSMNPs compared to the metal nanoparticles with larger sizes when their light absorbance is the same. Regardless of the nanoparticle size, the bulk-like decay process (i) of LSPR inevitably exists to generate heat to thermalize both the electrons and the lattices. The photothermal effect increases the temperature of the metal nanoparticles and the surrounding reaction solutions (or atmospheres), which is usually able to accelerate chemical reactions according to the Arrhenius equation.^14^ The simultaneous contributions of hot electrons and the photothermal effect to chemical reaction kinetics under photo-illumination^46,47^ makes it challenging to determine whether the high energy efficiency of hot electron generation in photoexcited QSMNPs can be translated to high energy efficiency in driving chemical reactions.

**Fig. 5:**
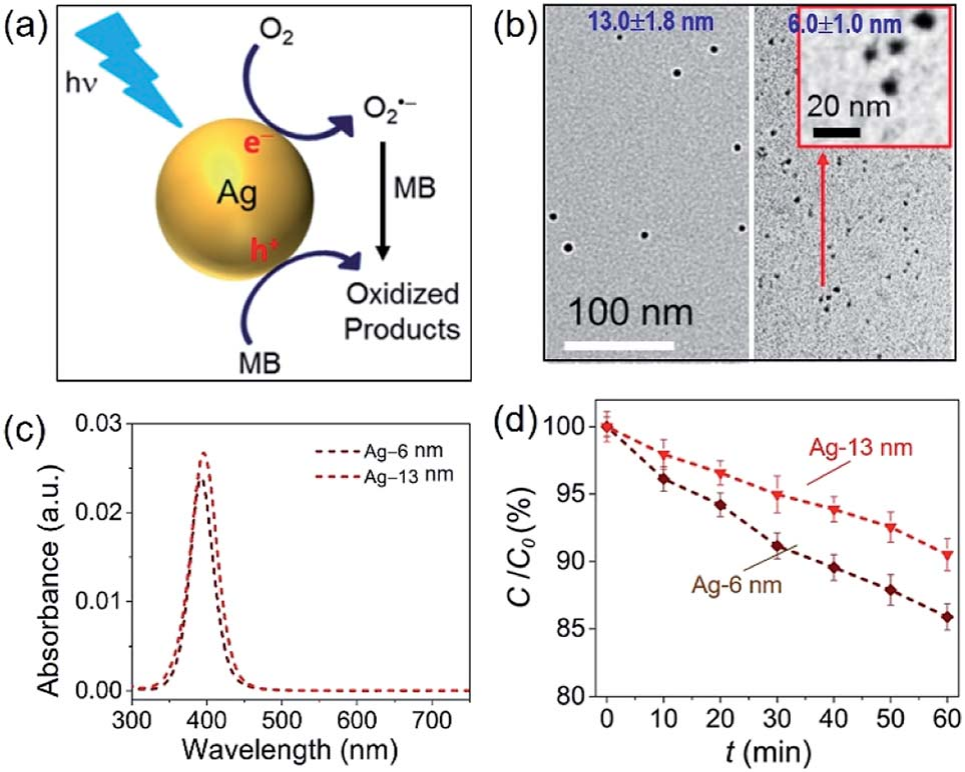
Oxidative degradation of methylene blue (MB) near the surfaces of photoexcited Ag nanoparticles under ambient aerobic conditions. (a) Schematic illustration highlighting the MB oxidation driven by the high-energy hot electrons and hot holes generated in the photoexcited Ag nanoparticles. (b) Typical TEM images of Ag nanoparticles with average diameters of (left) 13.0 nm and (right) 6.0 nm. The inset represents a blowup image of the smaller Ag nanoparticles. (c) Optical absorption spectra of the aqueous dispersions of the Ag nanoparticles shown in (b), highlighting the strong absorption peaks originating from LSPR. (d) Variation of MB concentration as the function of the photocatalytic reaction time, *t*, in the dispersions of the Ag naonparticles shown in (c) containing the same concentration of MB. The incident monochromatic light with a wavelength of 400 nm had an intensity of 20 mW cm^−2^. Adapted from ref. 15, AIP Publishing Copyright 2020.

The challenge can be tackled by performing hot-electron-driven chemical reactions in liquid solutions that maintain constant temperatures using external controls such as a large-volume water bath. Maintaining the constant temperature of reaction solutions excludes the possible contribution of the photothermal effect to the observed photocatalytic reaction kinetics, allowing us to more accurately evaluate the efficiency of hot electrons in driving chemical reactions. For example, oxidative degradation of methylene blue (MB) in aqueous solutions could be used as an exemplar reaction to study the energy efficiency of a hot-electron-driven reaction in the presence of Ag nanoparticles under photo-illumination.^15^ Since no MB degradation could occur in the dark even in the presence of the Ag nanoparticles, the oxidation of MB under photo-illumination is attributed to the hot-electron-driven reactions and hot-hole-driven reactions (if existing). As shown in Fig. 5a, the high energy of the photoexcited hot electrons in Ag nanoparticles drives these electrons to inject into the empty antibonding orbitals of the O_2_ molecules adsorbed on the Ag surface. The charge transfer process weakens the adsorption bonds to then detach the adsorbed O_2_ molecules, retaining the hot electrons to form superoxide radicals (O_2_˙^−^). Converting molecular oxygen (O_2_) to O_2_˙^−^ significantly enhances their oxidizing power to enable and promote MB oxidation.^48,49^ The leftover holes in the Ag nanoparticles can also oxidize MB to increase the kinetics of MB degradation further.^50,51^

In our most recent work,^15^ colloidal Ag nanoparticles with diameters of 13.0 ± 1.8 nm and 6.0 ± 1.0 nm (Fig. 5b) have been used to quantitatively compare the size-dependent efficiency of hot electrons in driving oxidative degradation of MB. The Ag nanoparticles of both 13.0 nm and 6.0 nm in diameter exhibit strong LSPR absorption in the ultraviolet (UV)-visible spectral region. The concentrations of Ag nanoparticle dispersions can be carefully adjusted to achieve the same optical absorbance around 400 nm for the differently sized Ag nanoparticles (Fig. 5c). To each dispersion of the Ag nanoparticles is added the same amount of MB (*i.e.*, 6 mg L^−1^) in the ambient atmosphere followed by illumination with monochromatic light of 400 nm, ensuring the same light absorption in the differently sized Ag nanoparticles. The MB molecules degrade faster in the presence of the smaller Ag nanoparticles of 6 nm diameter than the system containing the larger Ag nanoparticles of 13 nm diameter (diamonds *versus* triangles, Fig. 5d). The average turnover frequency (TOF) of a catalytic reaction is calculated using the amount of MB reacted over 60 min normalized against the total amount of available surface Ag atoms and the reaction time (*i.e.*, 60 min). Therefore, the value of TOF is independent of the surface area available in the differently sized Ag nanoparticles and represents a parameter for quantitatively evaluating the efficiency of hot-electron-driven MB degradation. The calculated TOF for the smaller 6 nm Ag nanoparticles is 0.058 s^−1^, which is 2.32 times of the TOF for the larger 13 nm Ag nanoparticles (*i.e.*, 0.025 s^−1^). The difference of TOF (*i.e.*, 2.32 times) is much larger than the difference of energy efficiency of hot electron generation (*i.e.*, 1.31 times) between the 6 nm Ag nanoparticles and the 13 nm Ag nanoparticles (Fig. 5d*versus*Fig. 4c). The comparison indicates that the unit of energy carried by the hot electrons in the 6 nm Ag nanoparticles is more efficient to drive the MB degradation reaction compared to the unit of energy carried by the hot electrons in the 13 nm Ag nanoparticles. Therefore, not only the high energy efficiency of generating hot electrons in the photoexcited Ag QSMNPs can be employed to drive chemical reactions but also the size-dependent reverse Coulomb blockade effect of the Ag QSMNPs favours efficient utilization of the energy carried by the hot electrons.

The data shown in Fig. 4 and 5 confirm that the unique size-dependent quantum phenomena in plasmonic QSMNPs are beneficial for achieving high efficiency of photo-to-chemical energy conversion mediated by hot electrons. However, the light absorption power of QSMNPs is weak even for the nanoparticles with strong LSPRs because the total light absorption (*Q*_total_) is proportional to the volume of the nanoparticles. The QSMNPs of compositions that do not exhibit LSPR in the visible spectral region, for example, most transition metals with good catalytic activities, exhibit even weaker light absorption power. Therefore, amplifying light absorption power in QSMNPs is crucial to increase the performance of photocatalytic hot-electron-driven chemical reactions upon a static illumination (*e.g.*, the sunlight). The optical absorption power (*W*) in a QSMNP is described by4
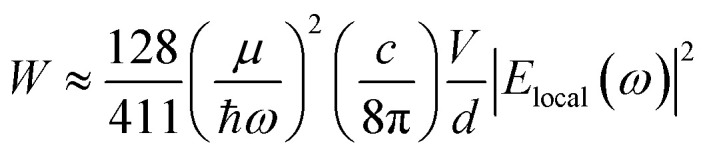
where ℏ, *c*, *ω*, and *μ*, are the reduced plank constant, speed of light, angular frequency of light, and Fermi energy of the corresponding metal, respectively, and *V* and *d* are the volume and diameter of the QSMNP, respectively.^13^ In addition to the volume of the QSMNP, the light absorption power is also proportional to the square of the local electric field (*E*_local_(*ω*)), in which the QSMNP locates. For a given incident light (*e.g.*, the sunlight), nanoscale optical antenna (or nanoantenna) have been explored to generate local electric fields much stronger than the field of the incident light by taking advantage of the unique light–nanoantenna interaction. When the catalyst QSMNPs are assembled near (or on) the surface of the nanoantenna, the QSMNPs can benefit from the enhanced local electric fields to improve the light absorption power in the QSMNPs.

Large plasmonic metal nanoparticles with strong light absorption can generate strong electric fields near their surfaces due to the photo-excited LSPR. The LSPR-enhanced local electric fields have been applied widely to amplify light scattering (*e.g.*, surface-enhanced Raman spectroscopy, or SERS),^52^ photoluminescence,^53,54^ and absorption (*e.g.*, surface-enhanced infrared absorption, or SEIRA).^55,56^ The enhanced local electric fields near the surface of plasmonic metal nanoparticles can reach a magnitude of up to 10^4^ times that of the incident light.^57^ Accordingly, appropriately placing QSMNPs around the surface of the antenna nanoparticles is effective in improving the optical absorption power of individual QSMNPs by 10^8^ times. For example, Antosiewicz *et al.* demonstrated the use of lithographically fabricated Ag nanodisks as light antennae to enhance the optical absorption power in Pd QSMNPs with sizes of less than 5 nm.^58^ It is worth pointing out that the Pd QSMNPs and the antenna Ag nanodisks have to be well separated by electrically insulating dielectric materials to avoid the charge exchange between them. Without the appropriate dielectric separation, the light absorption in the Pd QSMNPs cannot benefit from the enhanced surface electric fields of the Ag nanodisks. In the presence of a 5-nm Si_3_N_4_ layer between the Pd QSMNPs and the Ag nanodisks (thickness of 20 nm and diameter of 60 nm), the average optical absorption in the Pd QSMNPs could be as high as 10 000 times of that in the freestanding Pd QSMNPs. Colloidal plasmonic metal nanoparticles can also behave as light antennae to enhance optical absorption power in catalyst QSMNPs when appropriate approaches are developed to well separate these two types of metal nanoparticles with thin dielectric layers. For example, hybrid systems were synthesized using colloidal Al nanoparticles as light antennae on which QSMNPs of various transition metals (*e.g.*, Fe, Co, Ni, Ru, Rh, Pd, Ir, and Pt) were anchored.^59,60^ The Al nanoparticles and QSMNPs were separated by the native alumina layers spontaneously formed on the Al nanoparticles under ambient conditions. The enhanced optical absorption in the QSMNPs supported on the plasmonic metal nanoantennae is useful to improve the photocatalytic activity of the QSMNPs. For instance, the Pt QSMNPs of ∼5 nm in diameter assembled on silica-coated Ag nanoantennae (Ag@SiO_2_) exhibited photocatalytic activity toward CO oxidation higher than the freestanding Pt QSMNPs.^61^ Despite the success, the design of hybrid structures consisting of QSMNPs supported on plasmonic nanoantennae has some significant drawbacks to benefit hot-electron-driven chemical reactions maximally. The composition and thickness of dielectric layers between the QSMNPs and the plasmonic antennae have to be cautiously controlled to avoid the direct QSMNP-antenna charge exchange but to place the QSMNPs in the maximum local electric fields generated near the surface of the plasmonic antennae. The plasmonic antenna nanoparticles strongly absorb light to support the generation of locally enhanced electric fields, but the absorbed light eventually decays to heat in the antenna nanoparticles and cannot be absorbed by the QSMNPs to generate hot electrons. These drawbacks can be eliminated by using transparent dielectric antennae, on which the QSMNPs can be directly loaded without suffering the QSMNP-antenna charge exchange.

**Fig. 6:**
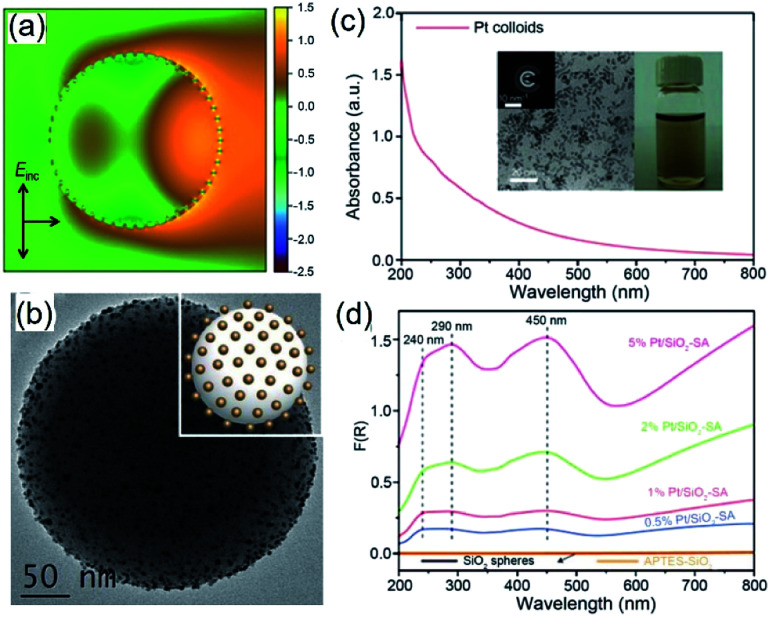
(a) Calculated intensity map of the near-field electric field (*E*) in and out of a silica sphere (with a diameter of 255 nm) decorated with 48 monodispered Pt QSMNPs (with a diameter of 4 nm). The map presents ln(|*E*/*E*_0_|^2^) in the plane of polarization for the Pt/silica hybrid particle. *E*_0_ represents the electric field of the incident light. (b) TEM image of typical Pt/silica hybrid particles synthesized through self-assembly of negatively charged Pt QSMNPs on the surface of positively charged silica nanospheres *via* strong electrostatic attractions. (c) Optical absorption spectrum of the aqueous dispersion of the freestanding Pt QSMNPs used for the synthesis of the Pt/silica hybrid particles shown in (b). The insets show the digital photograph of the dispersion and TEM image of the Pt QSMNPs. (d) DRS spectra of the Pt/silica hybrid particles with different Pt loadings: 0.5 wt%, 1 wt%, 2 wt%, and 5 wt%. The DRS spectra of silica nanospheres are also presented to highlight their inertness of absorbing light. Adapted from ref. 63, Macmillan Publishers Limited Copyright 2016.

Because of Fabry–Perot or Whispering Gallery resonances, spherical silica particles with the highest geometrical symmetry can generate enhanced electric fields near their surfaces.^62^ Depending on the size of silica spheres and resonance wavelength, the enhancement of |*E*_local_(*ω*)|^2^ near the silica surfaces can reach 10^3^ to 10^5^ to improve the optical absorption in the QSMNPs attached to the silica surface. For example, the rigorous finite-difference time-domain (FDTD) calculations of a silica sphere with a diameter of 255 nm decorated with Pt QSMNPs with a size of 4 nm upon light illumination with a wavelength of 450 nm reveals a distinct modal structure characteristic of Mie scattering resonances in a dielectric particle (Fig. 6a).^63^ When the Pt/silica structure is illuminated at a wavelength close to the scattering resonance frequency of the silica particles, the field maxima remain close to the Pt/silica interface. The enhanced local electric fields near the Pt/silica interface is capable of enhancing optical absorption power in the Pt QSMNPs. Zhang *et al.* synthesized the Pt/silica hybrid particles through self-assembly of negatively charged Pt QSMNPs on the surface of positively charged silica spheres due to strong electrostatic attraction (Fig. 6b).^63^ Loading the Pt QSMNPs on the silica spheres changes the optical absorption properties of the Pt QSMNPs significantly. As shown in Fig. 6c, the freestanding Pt QSMNPs with a size of 2–5 nm dispersed in an aqueous solution exhibit broadband optical absorption in both UV and visible spectral regions without showing any distinct absorption peak. The optical absorption of the silica spheres with a diameter of 225 nm is essentially zero in the range of 200–800 nm, which is presented in the absorption-sensitive diffuse reflectance spectroscopy (DRS) spectrum (Fig. 6d, orange curve). The DRS spectra of Pt/silica hybrid particles exhibit three distinct absorption peaks located around 240 nm, 290 nm, and 450 nm (Fig. 6d). The appearance of such distinct absorption peaks is remarkably different from the peakless absorption spectrum of the freestanding Pt QSMNPs, confirming the light antenna effect of the dielectric silica nanospheres in improving optical absorption power in the Pt QSMNPs. Using the dielectric silica spheres as light antennae is readily extended to enhance optical absorption power in QSMNPs of varying metals,^15,64–66^ semiconductor nanocrystals,^67^ metal/semiconductor composite NPs,^51^ and ultrafine oxide nanoparticles.^68^

**Fig. 7:**
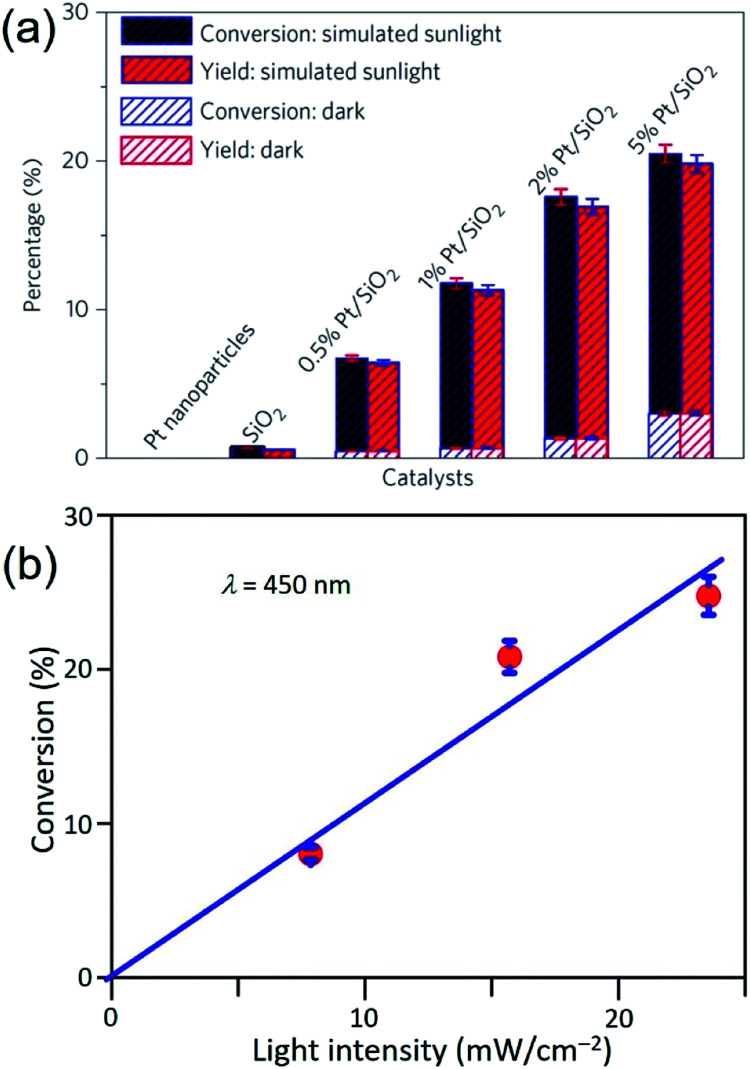
(a) Conversion percentage (blue) of benzyl alcohol (BA), and yield (red) of benzaldehyde (BAD) at room temperature under simulated sunlight or in the dark for 2 h in the presence of different particles: freestanding Pt QSMNPs, silica nanospheres, and Pt/silica hybrid particles with varying Pt loadings. (b) Conversion percentage of BA obtained over 5 wt% Pt/silica hybrid particles as a function of light intensity at room temperature under visible light irradiation centred at 450 nm for 10 h. The reactions were performed under the ambient aerobic conditions. Adapted from ref. 63, Macmillan Publishers Limited Copyright 2016.

Similar to the Ag QSMNPs presented in Fig. 5, shining the Pt QSMNPs with visible light is also expected to generate hot electrons to drive oxidation reactions, for example, aerobic selective oxidation of benzyl alcohol (BA) under ambient conditions. However, the freestanding Pt QSMNPs of Fig. 6c are almost photocatalytically inert toward oxidation of BA in the solvent of benzotrifluoride (BTF) under simulated sunlight at room temperature. The lack of measurable photocatalytic activity is ascribed to the weak light absorption in the freestanding Pt QSMNPs and poor colloidal stability. In contrast, the Pt/silica hybrid particles of Fig. 6b are photocatalytically active under the same conditions, showing the increase of activity with the loading content of Pt QSMNPs (Fig. 7a). For example, the conversion of BA reaches ∼20% with 97% selectivity of benzaldehyde (BAD) in the presence of Pt/silica hybrid particles with 5 wt% Pt loading when the system is illuminated with simulated sunlight for two hours. The difference of photocatalytic activity between the freestanding and the silica-supported Pt QSMNPs confirms the critical role of the spherical silica particles in enhancing the optical absorption in the Pt QSMNPs and improving the sequential oxidation reaction of BA. The influence of light intensity on the photocatalytic activity has been evaluated by using the monochromatic incident light centred at 450 nm, which is consistent with the absorption peak position of the Pt/silica hybrid particles. Fig. 7b shows that the photocatalytic activity is linearly dependent on the light intensity, confirming that the oxidation of BA is ascribed to hot-electron-driven processes rather than photothermal processes. The results indicate that hot-electron-driven photocatalytic chemistry more likely favours product selectivity, in contrast to conventional thermally driven chemical reactions. For instance, oxidation of different primary alcohols always led to high selectivity (>90%) of producing aldehydes by using the Pt/silica hybrid particles of Fig. 6b as the photocatalyst.^63^

In the scenario of hot electron injection into the empty states of adsorbate species, which do not retain the electrons, transient negative ions (TNIs) are formed with elevated potential to trigger surface chemical reactions while the electron lose energy and migrate back to the QSMNPs. Freund and Menzel studied the photoinduced desorption of NO from (NO)_2_ on Ag nanoparticles, in which the strongly nonthermal hot-electron distributions in the adsorbates enhance the normal desorption pathway.^69,70^ The photoinduced desorption cross section increases as the Ag nanoparticles decrease in size, showing a profound enhancement of 40 times on the Ag nanoparticles of 5 nm in diameter compared to the Ag(111) flat surface. The results confirm that reducing the size of QSMNPs is beneficial for hot-electron-driven surface chemistry regardless of whether the injected hot electrons are retained in the adsorbate species or not.^15,71,72^ Forming TNIs can also change the nuclear coordinate of adsorbate species to favour the surface reactions, for example, the (O_2_^−^)_ads_ TNIs exhibit longer O–O bond length compared with the adsorbed O_2_. The elongated O–O bonds increase the reactivity of adsorbed O_2_ to accelerate oxidation reactions on the photoilluminated Ag nanoparticles.^72^

## Photocatalytic surface chemistry of QSMNPs involving CID

The previous section mentions that chemically adsorbing reaction species on the surface of QSMNPs could trigger CID to directly decay plasmon energy into the metal/adsorbate interfacial states and avoid the intermediate creation of hot electrons in the QSMNPs. Suppose the energy transferred to the adsorbates through CID is high enough. In that case, electrons in the QSMNPs or the occupied metal–adsorbate complex states (*e.g.*, bonding orbitals) can be directly excited to the unoccupied states (*e.g.*, antibonding orbitals) of the adsorbates, activating the adsorbates to facilitate chemical reactions. The energy transfer from the excited plasmons to surface adsorbates could be ascribed to the LSPR-enhanced electric fields, which promotes a direct charge transfer within the metal–adsorbate complex at the metal/adsorbate interface.

The occurrence of plasmon-induced interfacial charge-transfer transition (PICTT) has been evidenced by the acceleration of anti-Stokes Raman scattering of MB chemically adsorbed on Ag nanocubes under excitation of a 785 nm laser beam.^73,74^ The PICTT charge excitation leads to an elevated population of excited vibrational modes compared with the equilibrated, thermalized system, which is responsible for the elevated anti-Stokes Raman scattering compared with that expected for a thermodynamically equilibrated system. The existence of PICTT direct charge excitation favours the degradation reaction of MB on the photoilluminated Ag nanocubes. The same mechanism was also observed in the promotion of catalytical O_2_ dissociation on photoilluminated Ag nanoparticles.^75^ In addition to metal/adsorbate systems, the PICTT direct charge excitation can also occur at the metal/semiconductor interface to promote electron transfer from plasmonic nanoparticles to the interfaced semiconductor nanostructures. For example, Wu *et al.* verified the PICTT mechanism in cadmium selenide (CdSe) nanorods with gold tips using transient absorption spectroscopy.^76^ The quantum efficiency of the PICTT process responsible for electron transfer from the Au tips to the CdSe nanorods could be high up to 24% and independent of excitation photon energy over ∼1 eV.

Strong-enough chemical adsorption of molecules on small-enough QSMNPs could form highly hybridized adsorbate–metal states that result in the new optical absorption band in the adsorbate–metal system. In this case, direct photoexcitation of the hybridized adsorbate–metal states is likely to dominate catalytic photochemistry. For example, selective photocatalytic oxidation of CO has been observed on the Pt QSMNPs with sizes less than 5 nm.^77^ The dependence of reaction rate on the wavelength of incident light exhibits the same profile as the newly emerged optical absorption band, indicating that direct photoexcitation of the CO–Pt hybridized states is responsible for the catalytic activity.

## Remarks and perspectives

Electrons in “quantum-sized metal nanoparticles” with size in the range of 2–10 nm exhibit size-dependent body quantum behaviour (*i.e.*, photoexcited LSPR) and strong surface quantum behaviours (*i.e.*, generation of high-energy hot electrons and the Coulomb blockade effect). The coincident existence of strong quantum phenomena both in the body and the surface of QSMNPs results in the strong interdependence and synergy of light absorption and surface chemistry. The strong synergy shines a light on the promise of using the QSMNPs made of various transition metals as a new class of efficient photocatalysts for selective chemical transformations. The examples presented in the previous section highlight the role of QSMNPs in improving the reactivity of reactant species and selectivity of products through the mediation of the photoexcited hot electrons and the complimentary hot holes. Moreover, the involvement of high-energy hot electrons may enable the occurrence of thermodynamically endergonic reactions since the energy carried by the hot electrons could lift the total Gibbs energy of the system, including the reactants and the hot electrons, to be higher than the Gibbs energy of the products. Because electrons do not have a chemical identity, hot-electron-driven chemical reactions are ready to be extended to photoexcited QSMNPs made of a broad range of transition metals that have been widely used as catalysts for important reactions.

The emergence of using QSMNPs as photocatalysts will help plenty of research opportunities to advance. However, extraordinary challenges are ahead to prevent from achieving the full capacity of hot-electron-driven chemical transformations on QSMNPs. For instance, the large-area surfaces of QSMNPs with sizes of 2–10 nm are usually complicated. The physical and chemical specifications of these surfaces are crucial to interact with the surface adsorbate molecules. Systematically characterizing the crystalline orientations and crystalline perfections (or defects) of the surfaces of QSMNPs is inevitably important to help comprehensively understand the surface specifications as a function of the particle size and fabrication methods. Aberration-corrected TEM imaging represents an advanced technique to characterize nanoparticle surfaces at atomic levels, as shown in the example in Fig. 3a. The availability of surface specifications will be helpful to determine the adsorption configurations of the surface adsorbates, which are crucial to influencing the interaction strength between the adsorbate molecules and the surface metal atoms and the charge exchange dynamics in the course of photocatalysis. Controlling the flow direction of high-energy hot electrons generated in QSMNPs to the target chemical bonds of the surface adsorbate species is promising to improve product selectivity of photocatalytic reactions. Hot electrons preferably inject into the empty antibonding orbitals with a high local density of states (LDOS) and lower energy to selectively activate the corresponding chemical bonds in the adsorbate species. Therefore, appropriate adsorption configurations of the reactant molecules on the QSMNPs is essential to determine the flow dynamics and flow direction of hot electrons, influencing the kinetics of the sequential chemical reactions. Theoretical modelling and calculations, such as density functional theory (DFT) will play an important role in helping comprehensively understand the adsorption configuration of reactant molecules on metal surfaces and the LDOS profiles. Since surface atoms represent a significant portion of atoms in QSMNPs, the strong interaction between the surface metal atoms and adsorbate species can influence the electronic structures (*e.g.*, LDOS profiles, dislocation of electrons, *etc.*) of the inner metal atoms in the QSMNPs. Using the actual volume of QSMNPs rather than a small surface slice to build the theoretical model becomes necessary to calculate the QSMNP/adsorbate systems, although the calculation cost could be expensive currently. The accurate calculation results will provide guideline feedback for designing the optimum QSMNPs with the appropriate surfaces to adsorb reactant molecules in the desirable configurations, which favour the flow of hot electrons into the preferable chemical bonds.

Moreover, both the short distance from the surface metal atoms to the target chemical bonds and the strong adsorption are favourable for the rapid injection dynamics of hot electrons to improve the surface reaction kinetics. Relaxation of photoexcited plasmons in a QSMNP with adsorbate reactant molecules involves many sequential and interdependent pathways to dissipate energy at very short time scales (ps–fs). The study of these fundamental processes has emerged due to not only the importance and promise of hot-electron-driven photocatalysis but also the technological advancement and availability of characterization facilities. For instance, ultrafast transient absorption spectroscopy with the use of a femtosecond laser pump and probe is widely employed to study the time-resolved generation and retention of hot electrons in metal nanoparticles, in particular, plasmonic metal nanoparticles with characteristic strong optical absorption.^78^ Time-resolved X-ray absorption spectroscopy built on the large synchrotron facilities became available recently to study the transient change dynamics of oxidation states of the QSMNPs upon loss of hot electrons when the QSMNPs do not exhibit well-defined LSPR absorption peaks. The time-resolved X-ray absorption spectroscopy is also capable of proving the transient states of adsorbate species upon injection of hot electrons when the low-energy (soft) X-rays are used as the probe, providing the transition dynamics of adsorption geometry and bonding strength of the adsorbates.^79,80^ Transient infrared (IR) absorption spectroscopy provides an additional technique to probe the dynamics of transient states of adsorbate species that are responsible for IR absorption.^81^ Although fully understanding the transient dynamics of hot electrons involved in hot-electron-driven photocatalytic reactions is significantly challenging, researchers are continuously advancing the characterization techniques to enable the study on the relaxation processes of the high-energy hot electrons with high resolution in both spatial and temporal space. The foreseeable research activities will eventually bring useful information together to help understand the sequential quantum processes related to the relaxation and flow of hot electrons. This understanding will, in turn, guide the design of QSMNPs that are favourable for photocatalysis with high photo-to-chemical conversion efficiency and product selectivity.

Because of the small size of QSMNPs, it is challenging to synthesize well-dispersed QSMNPs and maintain their stability and maximum surface areas under working conditions. The QSMNP/silica design shown in Fig. 6 represents a promising strategy to enable good dispersity and stability of the Pt QSMNPs even when they are used for photocatalytic oxidation of BA. This example highlights that high-refractive-index dielectric nanospheres may represent a unique class of support for QSMNPs. Loading (anchoring) QSMNPs on the dielectric nanospheres with a size of hundreds of nanometers can effectively prevent movement and agglomeration of the QSMNPs to maintain their dispersity on the surface of the dielectric nanospheres. Moreover, the strong light scattering resonances of the dielectric nanospheres produce enhanced electric fields near their surfaces to maximize the optical absorption power of the anchored QSMNPs, benefiting the generation of hot electrons as well as the sequential hot-electron-driven chemical reactions. Previous work has demonstrated great success in the self-assembly of the monodispersed QSMNPs of noble metals on the surfaces of dielectric nanospheres through strong electrostatic attractions, resulting in the formation of the desirable hybrid particles (see the example shown in Fig. 6b). As for the QSMNPs made of transition metals that are not stable under ambient conditions, the multistep protocol involving the impregnation of ionic metal precursors and the following reduction could be used to synthesize the target hybrid particles. The first step is to synthesize dielectric nanospheres with the appropriate sizes and surface properties (*e.g.*, porosity, surface functional groups, surface charges, *etc.*) that are suitable for driving impregnation of ionic metal precursors. Accomplishing the synthesis of stable QSMNPs on dielectric nanospheres can benefit from the positive knowledge in the vast literature that reported the use of the impregnation method for the synthesis of metal/oxide composite nanoparticles.^82–84^ It is worth pointing out that the crystalline type (*e.g.*, terraces, edges, corners, steps, kinks, twin boundaries, and vacancies) and oxidation state of surface metal atoms of QSMNPs may vary under the reaction conditions to influence their catalytic activity.^85,86^ The influence can be either positive or negative toward the promotion of catalytic activity, which adds one more dimension of complexity of designing/synthesizing stable and efficient QSMNP photocatalysts. When the working conditions are too harsh (*e.g.*, high temperatures, reactive environments) to maintain the stability of QSMNPs, an additional inert mesoporous layer with an appropriate thickness can be overgrown outside the QSMNPs to improve the stability of the QSMNPs. The diffusion of reaction species to (and away from) the surface of QSMNPs is still feasible through the pores of the mesoporous layer.

In summary, the combined future efforts in materials synthesis, advanced characterization, and theoretic modelling and calculations will provide a solid foundation to explore the full potential of QSMNPs in photocatalytic reactions. As indicated in the results presented in Fig. 5 and 7, the hot-electron-driven photocatalytic reactions on QSMNPs show promise in simultaneously accelerating reaction kinetics and improving product selectivity. This promise overcomes the major challenge in thermally driven catalysis, in which reaction kinetics is improved with an increase of temperature, but the product selectivity is usually sacrificed. Although the photothermal effect in metal nanoparticles is inevitable, the percentage of photoenergy converted to thermal energy is significantly suppressed in QSMNPs (Fig. 4) to maximize the photo-to-chemical energy conversion through the hot-electron-driven pathways. In addition, the undesirable photothermal effect can be eliminated with external controls, such as immersing the reactors in large-volume oil/water baths maintained at room temperature, to signify the merits of hot-electron-driven photocatalysis further. Therefore, QSMNPs made of appropriate metals loaded on appropriate dielectric nanospheres represent a brand-new class of photocatalysts to explore chemical synthesis with high product selectivity.

## Conflicts of interest

There are no conflicts to declare.

## Supplementary Material
